# Boosted Photocatalytic Activities of Ag_2_CrO_4_ through Eu^3+^-Doping Process

**DOI:** 10.1021/acsomega.4c02683

**Published:** 2024-08-07

**Authors:** Josiane C. Souza, Samantha C. S. Lemos, Marcelo Assis, Carlos H. M. Fernandes, Lara K. Ribeiro, Yeison Núñez-de la Rosa, Márcio D. Teodoro, Lourdes Gracia, Juan Andrés, Lucia H. Mascaro, Elson Longo

**Affiliations:** † CDMF, Federal University of São Carlos (UFSCar), São Carlos 13565-905, Brazil; ‡ Department of Physical and Analytical Chemistry, University Jaume I (UJI), Castelló 12071, Spain; § Department of Chemistry, Federal University of São Carlos (UFSCar), São Carlos 13565-905, Brazil; ∥ Faculty of Engineering and Basic Sciences, Fundación Universitaria Los Libertadores, Bogotá 111221, Colombia; ⊥ Department of Physics, Federal University of São Carlos (UFSCar), São Carlos 13565-905, Brazil; # Department of Physical Chemistry, University of Valencia, Valencia 46010, Spain

## Abstract

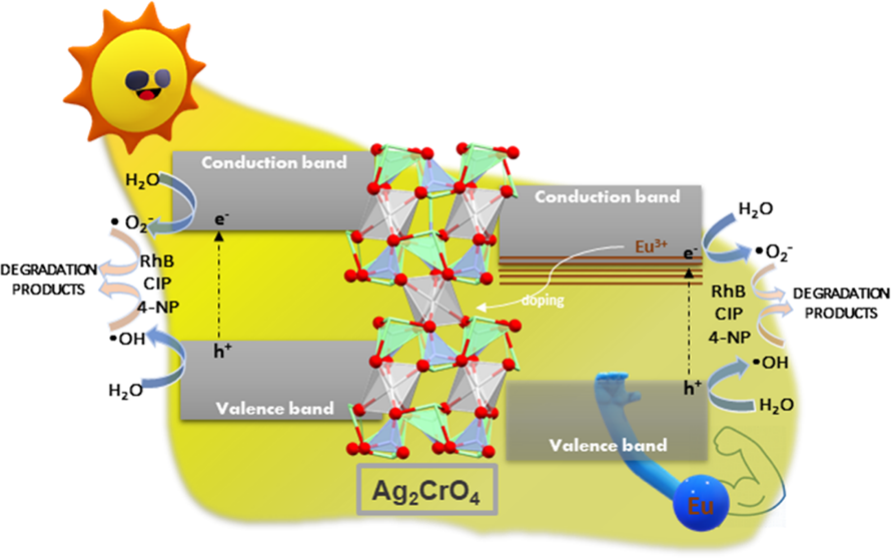

Ag_2_CrO_4_ is a representative member of a family of Ag-containing semiconductors with highly efficient visible-light-driven responsive photocatalysts. The doping process with Eu^3+^ is known to effectively tune their properties, thus opening opportunities for investigations and application. Here, we report the enhancement of the photocatalytic activity and stability of Ag_2_CrO_4_ by introducing Eu^3+^cations. The structural, electronic, and photocatalytic properties of Ag_2_CrO_4_:*x*Eu^3+^ (*x* = 0, 0.25, 0.5, 1%) synthesized using the coprecipitation method were systematically discussed, and their photodegradation activity against rhodamine B (RhB), ciprofloxacin hydrochloride monohydrate (CIP), and 4-nitrophenol (4-NP) was evaluated. Structural analyses reveal a short-range symmetry breaking in the Ag_2_CrO_4_ lattice after Eu^3+^ doping, influencing the material morphology, size, and electronic properties. XPS analysis confirmed the incorporation of Eu^3+^ and alteration of the surface oxygen species. Furthermore, photoluminescence measurements indicated that the doping process was responsible for reducing recombination processes. The sample doped with 0.25% Eu^3+^ exhibited superior photocatalytic performance compared to pure Ag_2_CrO_4_. Scavenger experiments revealed an increase in the degradation via ^•^OH reactive species for the sample doped with 0.25% Eu^3+^. DFT calculations provided atomic-scale insights into the structural and electronic changes induced by the Eu^3+^ doping process in the Ag_2_CrO_4_ host lattice. This study confirms that Eu^3+^ doping alters the band structure, enabling different degradation paths and boosting the separation/transfer of photogenerated charges, thereby improving the overall photocatalytic performance.

## 1 Introduction

The rapid and uncontrolled development of human societies has led to a current challenge in managing chemical waste improperly dumped.^1−3^ Large industries, as well as agribusiness, stand out as major sources of toxic chemical waste. The increasing lack of legislation contributes to the accumulation of this problem. Specifically, wastewater poses a serious concern, as improper treatment can result in significant impacts on society and the environment due to its toxic, mutagenic, and carcinogenic characteristics.^4^ Among the broad class of organic pollutants, agrochemicals and antibiotics can cause serious issues, including increased bacterial resistance and heightened toxicity, even at low concentrations.^5^

One of the increasingly utilized antibiotics is ciprofloxacin hydrochloride monohydrate (CIP), belonging to the class of fluoroquinolones, which is widely used to treat bacterial infections in various body systems. CIP can be found in wastewater in the range of 1 × 10^–6^ to 1 ppm, potentially causing serious harm when consumed excessively.^6^ Among agrochemicals, one of the foundational molecules for many of these pollutants is 4-nitrophenol (4-NP), which can cause blood disorders and eye and skin irritation, as well as kidney, liver, and central nervous system damage in humans and other animals.^7,8^ Brazilian legislation establishes a limit for some 4-NP derivatives in wastewater of up to 500 ppb, while US legislation restricts concentrations above 10 ppb in wastewater.^9,10^ Due to the significant harmful potential of human and environmental exposure, these two molecules serve as excellent study models to assess the potential wastewater removal through chemical and physical processes.

The widespread use of wastewater products demands proper end-of-life management to reduce environmental threats. Photocatalysis driven by inorganic semiconductors offers a clean and sustainable approach to carry out chemical conversions under ambient conditions due to its low cost, reusability, and ability to harness sunlight to initiate the photodegradation process of organic molecules, potentially leading to their complete mineralization, i.e., transformation into CO_2_ and H_2_O.^11−15^ Meanwhile, for this type of photocatalysis to be viable, the focus should be on developing semiconductors that absorb light in the visible region and can be obtained through simple synthesis methods. Among the Ag-based semiconductors, Ag_2_CrO_4_ stands out for its broad UV–vis and visible absorption spectrum, excellent photogenerated carrier transfer to generate efficiently reactive oxygen species (ROS), and broad UV–vis and visible absorption spectrum, providing an advantage in photocatalytic applications.^16−18^ However, like Ag-based semiconductors, this material is prone to photocorrosion processes, which significantly reduce its stability and recyclability, thereby limiting its use as a photocatalyst.^19^

One strategy to enhance the efficiency and stability of semiconductors is through doping with heteroatoms. Pinatti et al. doped Ag_2_CrO_4_ with Zn^2+^, assessing its photocatalytic properties for the photodegradation of Rhodamine B (RhB) and its antimicrobial characteristics.^20^ It was observed that even at low concentrations, Zn^2+^ doping increased visible light absorption by introducing new intermediate levels in the band gap region. This, along with inducing morphological modifications, contributed to enhanced stability against consecutive photodegradation cycles. Among the heteroatoms used for doping, rare earth cations have been proven to intensify photocatalytic performance by improving structural and electronic properties, modulating energy band structures, and facilitating the production and separation of photogenerated electron–hole pairs during the photocatalytic reaction.^21−23^ In particular, Eu^3+^ has been extensively employed as a dopant in other inorganic semiconductors, achieving higher efficiencies and imparting greater stability to the semiconductor.^22−27^

Here, we report the synthesis of Ag_2_CrO_4_:*x*Eu^3+^ (*x* = 0, 0.25, 0.5, 1%) by the coprecipitation method for photocatalytic application. Characterization of the as-synthesized samples was performed by X-ray diffraction (XRD), Raman and X-ray photoelectron spectroscopies (XPS), UV–vis diffuse reflectance spectroscopy (DRS), field emission scanning electron microscopy (FE-SEM), and photoluminescence (PL) spectroscopy. Their photocatalytic performances were examined by degrading the RhB, CIP, and 4-NP under visible light illumination, and the obtained findings were discussed in detail. Scavenger experiments were performed to identify the active species in the degradation process, and catalyst recycling was analyzed to assess the stability of the photocatalyst. To complement experimental results, density functional theory (DFT) calculations were performed to gain deep insight into the structural and electronic effects of the Eu^3+^ doping process. The paper is organized as follows: in the next section, the results are presented and discussed. Finally, the conclusions are presented in Section 3. Detailed information about the materials and methods, characterization techniques, computational methods, and model systems can be found in the Supporting Information.

## 2 Results and Discussion

### 2.1 Structural and Electronic Characterization

**Figure 1:**
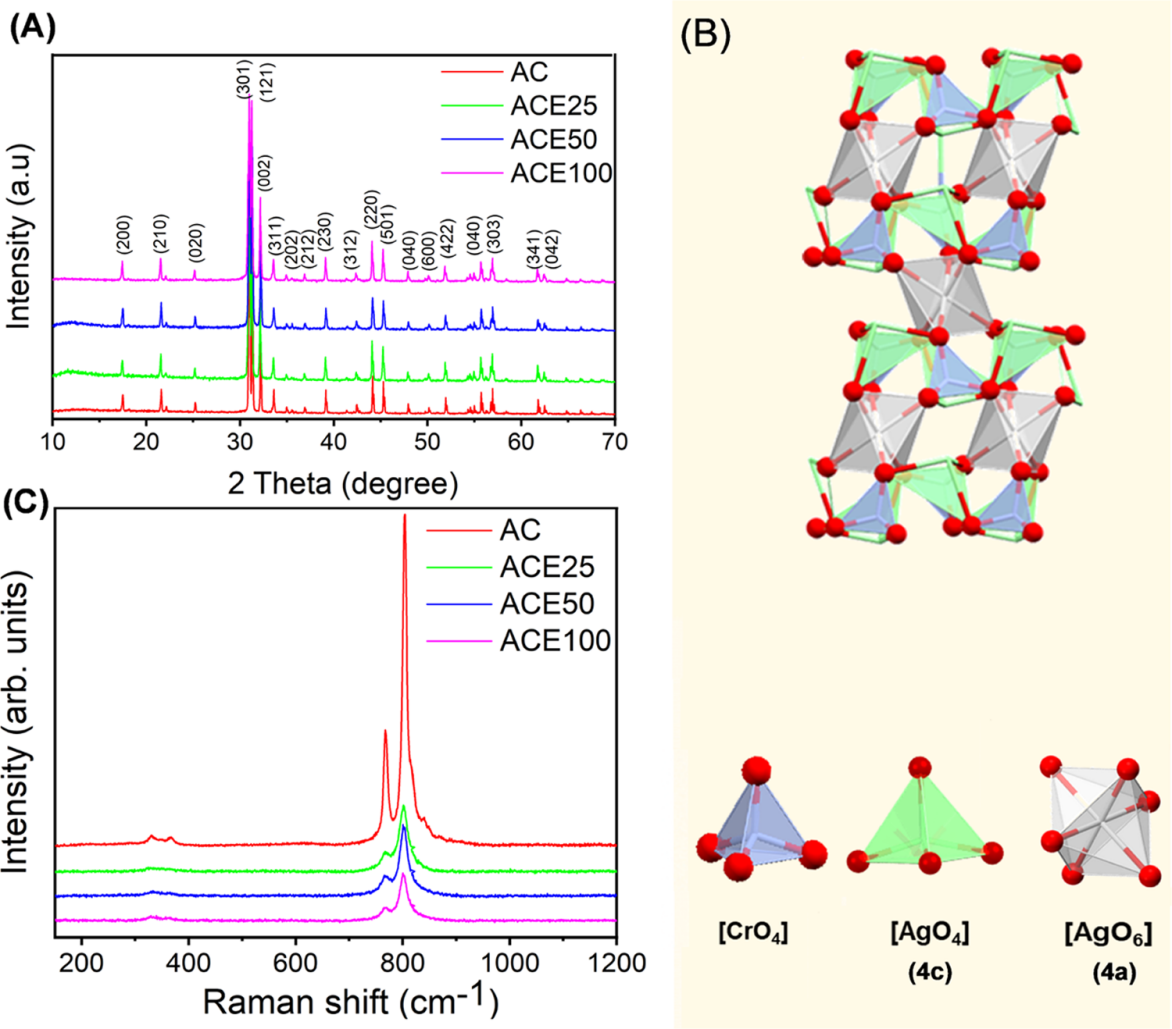
(A) XRD patterns of the of the as-synthesized Ag_2_CrO_4_:*x*Eu^3+^ (*x* = 0, 0.25, 0.5, 1%) samples. (B) 3D representation of Ag_2_CrO_4_ structure. The local coordination of Cr^6+^ and Ag^+^ cations corresponding to [CrO_4_], [AgO_4_], and [AgO_6_] clusters, are highlighted. (C) Raman spectra of the samples.

To analyze the modifications induced by Eu doping in the Ag_2_CrO_4_ matrix over both long and short ranges, XRD and Raman spectroscopy measurements were conducted and are presented in Figure 1. For clarity, Ag_2_CrO_4_:*x*Eu^3+^ (*x* = 0, 0.25, 0.5, 1%) were denoted as AC, ACE25, ACE50, and ACE100, respectively. According to the XRD results in Figure 1A, all of the samples exhibit a single orthorhombic structure, consistent with reference number 252779 in the Inorganic Crystal Structure Database (ICSD).^28^ The sharp peaks depicted in the diffractograms indicate the crystalline nature of the system. Peaks attributed to second-phase formation or impurities were not observed. For a better understanding of the influence of Eu^3+^ doping on the crystal lattice of Ag_2_CrO_4_, Rietveld refinements were performed on the samples. The lattice parameters (*a*, *b*, *c*), as well as the quality coefficients of the refinement (χ^2^, *R*_p_, *R*_wp_) are listed in Table S1, while the Rietveld refinement plots are presented in Figure S1A–D. The lattice parameters obtained from the ACE25, ACE50, and ACE100 samples did not differ significantly from those of the AC sample. Present lattice parameters are in good agreement with previously experimental values (Figure S1 and Table S2). The Ag_2_CrO_4_ shows an orthorhombic unit cell composed of distorted clusters of [CrO_4_], [AgO_4_], and [AgO_6_] with space group *Pnma*. In this structure, two types of local coordination exist for Ag^+^, [AgO_6_] and [AgO_4_] clusters, corresponding to Wyckoff positions 4a and 4c, respectively (Figure 1B).

The Raman spectra of the as-synthesized samples are displayed in Figure 1C. According to group theory, Ag_2_CrO_4_ has 36 active Raman modes, which can be easily altered due to the crystallinity of the system.^20,29^ An analysis of the results shows that for the AC sample, two Raman peaks with high intensity appear, 767 and 807 cm^–1^, corresponding to the A_g_ mode, which are associated with the symmetric stretching vibrations of Cr–O bond in the [CrO_4_] clusters.^30^ Three peaks at 320, 390, and 840 cm^–1^ with a very weak intensity can be sensed, which are associated with B_3g_ and B_2g_ modes, corresponding to the bending modes of the [CrO_4_] cluster. The Eu^3+^ doping process provokes the disappearance of these three peaks with weak intensity, while the intensity of the two A_g_ peaks decreases. This behavior confirms the short-range order of the samples and demonstrates that the small amount of Eu^3+^ cations was not sufficient to give rise to strong structural changes of the host lattice. A theoretical study of the calculated Raman spectra on model systems of Ag_2_CrO_4_ and Eu^3+^doped systems in 4a and c is presented and discussed in the Supporting Information (see Figure S2). The increase of the short-range disorder for the Eu^+^-doped Ag_2_CrO_4_ samples was verified by the analysis of the full width at half-maximum (FWHM) of the most intense peak (807 cm^–1^): an increase from 8.7 cm^–1^ in the pure sample to values greater than 16.0 cm^–1^ in the doped samples was observed (Table S3).

**Figure 2:**
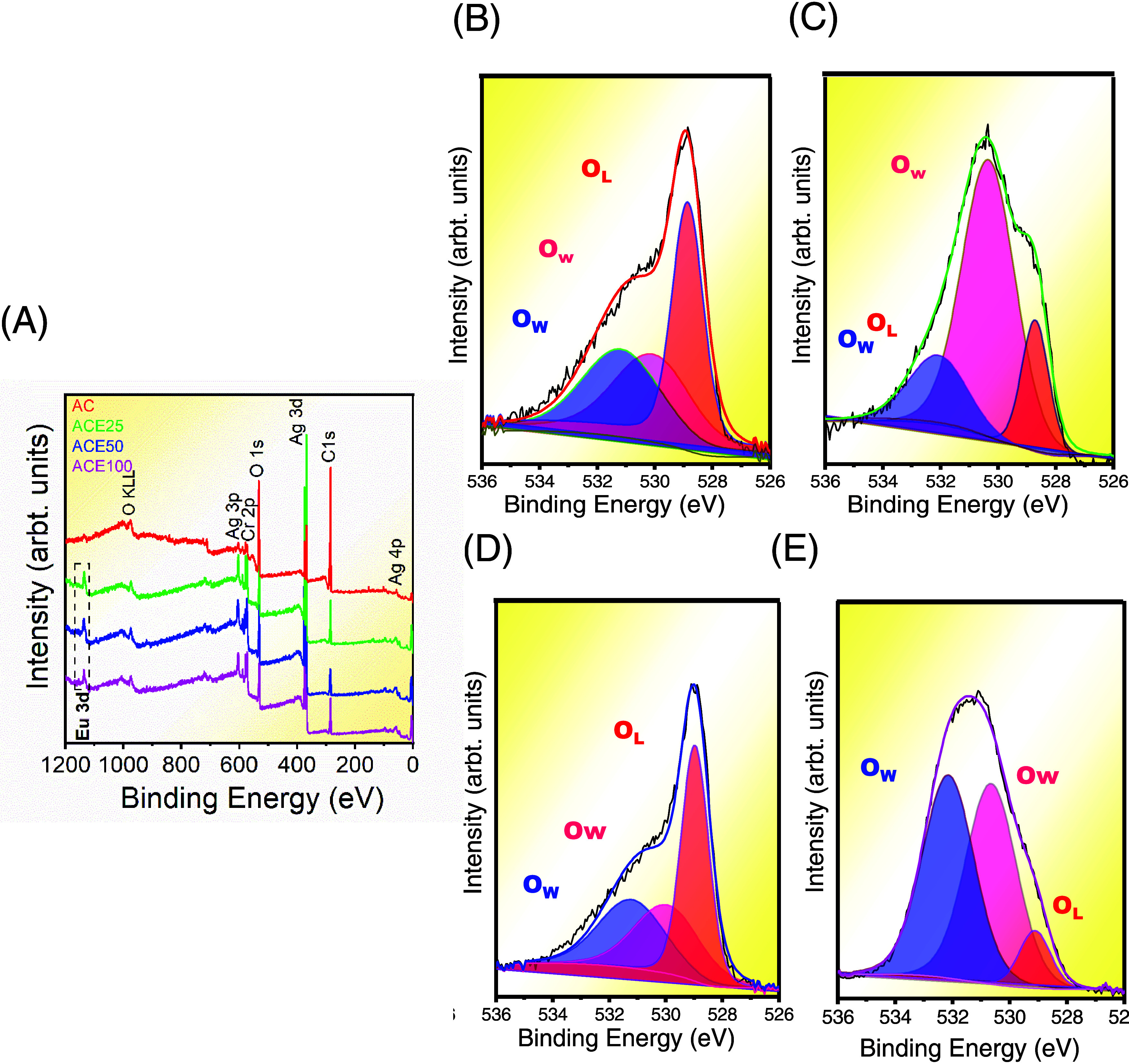
(A) Survey XPS spectra of the samples. High-resolution XPS spectra of O 1s for the indicated samples. O_W_ = adsorbed water/hydroxyl; O_L_ = lattice oxygen. (B) AC, (C) ACE25, (D) ACE50, and (E) ACE100.

Figure 2A shows the survey of XPS spectra with different binding energies of the Ag_2_CrO_4_ and Eu^3+^-doped Ag_2_CrO_4_ samples. The binding energies at 603.12,^31^ 367.15,^32^ and 61.64 eV are assigned to Ag 3p, Ag 3d, and Ag 4p levels, respectively, while at 572.27^33^ and 532.09 eV,^34^ they correspond to Cr 2p and O 1s levels, respectively. The appearance of Eu 3d (∼1144 eV)^35^ confirms the Eu^3+^ doping process at Ag_2_CrO_4_. Figure 2B–E depicts the high-resolution XPS spectra of the O 1s core level. It is important to note that assigning XPS spectra to O vacancies in systems with different transition metals is controversial, as reported by Idriss.^34^ The broad peak observed in the spectrum is characteristic of multiple oxygen states, and therefore can be deconvolved into two types of oxygen with different chemical shifts around 529, 530, and 532 eV, attributed to the oxygen crystal lattice (O_L_) of Ag_2_CrO_4_, and adsorbed water/hydroxyl species (O_W_), respectively.^19,34^ In all samples, the contributions of O_L_ and O_W_ can exhibit random intensities and dislocations, where each doping concentration affects the surface composition in a particular way.

**Figure 3:**
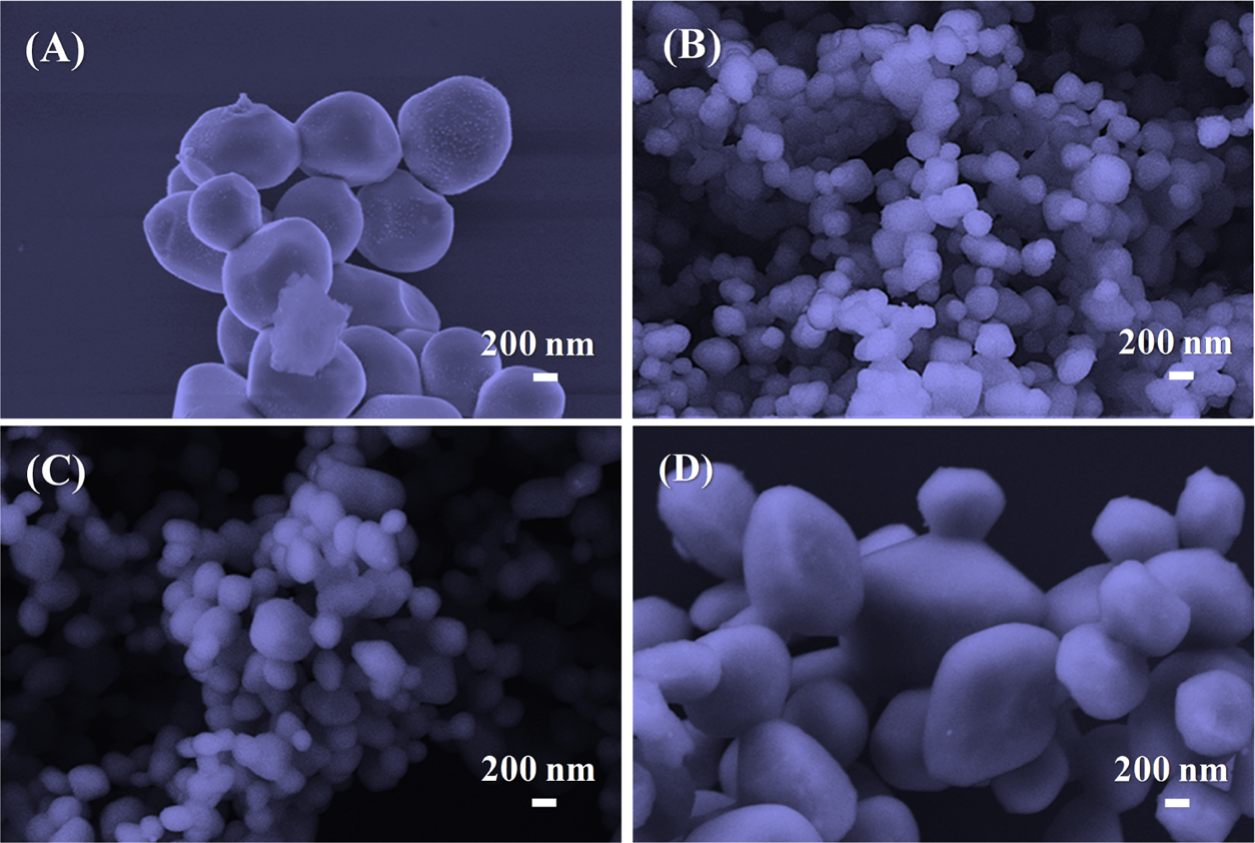
FE-SEM images of samples (A) AC, (B) ACE25, (C) ACE50, and (D) ACE100.

Figure 3 shows the FE-SEM images of the Ag_2_CrO_4_ samples. The particles present like-flattened spheres with average sizes of 1048 298, 308, and 710 nm for the AC, ACE25, ACE50, and ACE100 samples, respectively. Related morphologies were obtained previously by our research group.^18,20,36^

Figure S3A displays the DRS analysis of the samples. The absorption edge for all samples was determined to be approximately 650 nm, indicating a high absorption of visible light. Similar to many semiconductors, calculations on Ag_2_CrO_4_ revealed that one of its notable characteristics is being a semiconductor with an indirect band gap.^19,28,36,37^Figure S3B–E show the energy of band gap (E_gap_) for all samples. It is noteworthy that the E_gap_ of pure Ag_2_CrO_4_ was 1.69 eV, and no significant variations were detected in the Eu^3+^-doped samples.

PL spectra are presented in Figure S4. Three emissions appear in the red/near-infrared region (806, 876, and 971 nm), which can be associated with the presence of multilevels within the band gap region and multiphotonic processes generated from defects in the Ag_2_CrO_4_ structure, favoring the recombination of charge carriers.^20,36,38−40^ The characteristic emission lines of Eu^3+^ transitions are not observed for the Eu^3+^-doped samples due to their dark brown color, which rapidly absorbs all radiation that could be produced by this cation. A sharp reduction in the intensity of the PL emission is observed in the Eu^3+^-doped samples, with a more pronounced decrease in the ACE50 and ACE100 samples. The substitution of Eu^3+^ cations at the Ag_2_CrO_4_ lattice provokes the formation of Ag^+^ vacancies (V_Ag_), which leads to decreased PL intensity via luminescence quenching. This is an indication that the recombination of photogenerated charge pairs in the doped samples is lower, which may result in a higher photocatalytic performance for these samples.^41^

### 2.2 Photocatalysis

**Figure 4:**
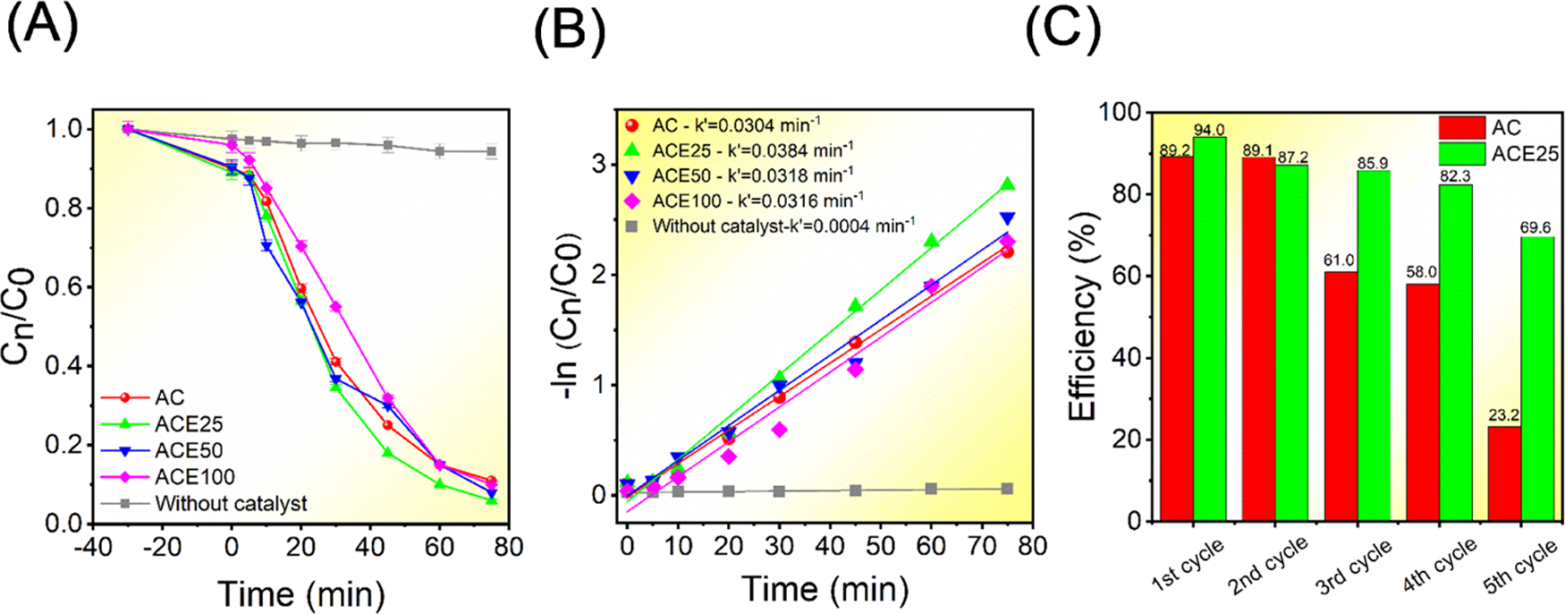
(A) Relative concentration of RhB (*C_n_*/*C*_0_). (B) Reaction kinetics of RhB degradation, −ln(*C_n_*/*C*_0_) vs time. (C) Photocatalytic recycling for RhB degradation over the ACE25 and AC samples.

In this work, the photodegradation of RhB under visible light was initially conducted to identify the best-doped sample for further work on the photodegradation of CIP and 4-NP (Figure 4). It is possible to observe in Figure 4A a slight increase in the RhB photodegradation activity for the ACE25 sample compared to that of the pure AC sample. To further analyze this behavior, the kinetics of the photodegradation process were examined using the pseudo-first-order kinetic model, which showed a good fit for all samples. In comparison to the pure AC sample, all samples doped with Eu^3+^ exhibited a slight increase in the rate constant (k value), changing from 0.0304 min^–1^ for the AC sample to 0.0384, 0.03018, and 0.0316 min^–1^ for the ACE25, ACE50, and ACE100 samples, respectively (Figure 4B). Additionally, the stability under successive cycles of photodegradation was evaluated to compare the effects of photocorrosion between the pure AC and ACE25 samples (Figure 4C). For the AC sample, a sharp decline in photocatalytic efficiency was observed after the second cycle, reaching 23.2% efficiency by the fifth cycle. In contrast, the Eu^3+^-doped sample showed a significant reduction after the fourth cycle, with efficiency reaching 69.6% by the end of the fifth cycle. To explain this difference, we suggest that ACE25 is capable of decreasing the recombination processes of the photogenerated electron–hole pairs to a major extent compared to AC, thus increasing the efficiency of the photodegradation along the recycling runs.

The AC and ACE25 samples, collected after five cycling runs, were characterized by using XRD analysis, FE-SEM images, and PL spectroscopy. An analysis of the XRD results renders the presence of a small peak at 2θ ≅ 38.2° (Figure S5). This peak with low intensity can be associated with a small content of cubic Ag^0^ (ICSD number 41690),^42^ whereas these samples before the degradation process do not display Ag^0^ peaks. Similarly, Zhang and Ma^43^ have reported that the amount of Ag^0^ is increased after the photodegradation process of Ag/Ag_3_VO_4_/AgIO_3_ heterostructure. The FE-SEM images of the samples after the successive cycles show that the particle shapes were preserved after the degradation process, maintaining like-flattened sphere structures (Figure S6). The average particle size was 746 nm for the used AC sample and 287 nm for the used ACE25 sample. This corresponds to a reduction in average particle size of 39% for the used AC sample and 4% for the used ACE25 sample compared to the respective samples before the photodegradation process (see Figure 3).

**Figure 5:**
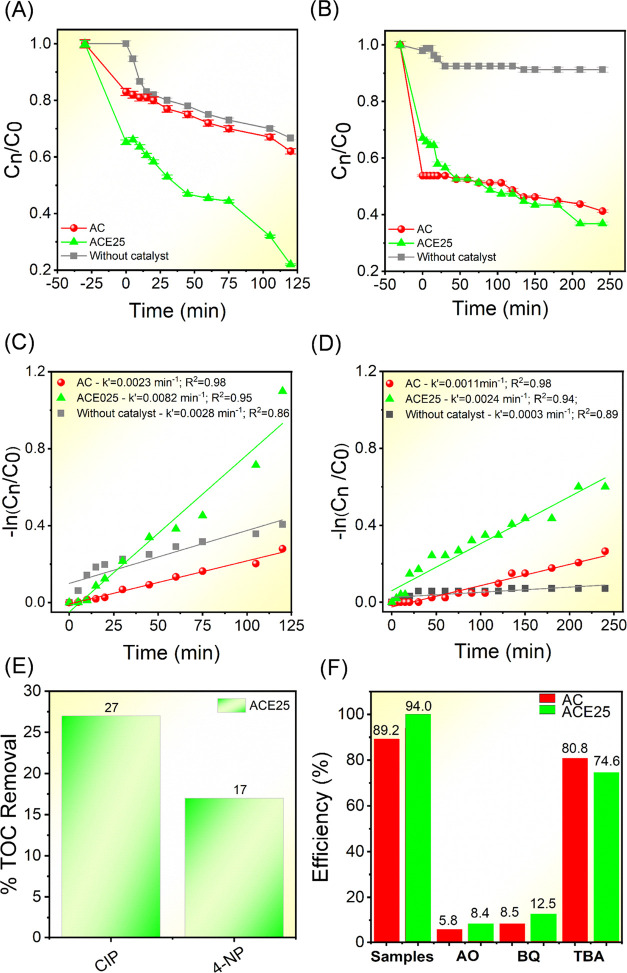
Photodegradation profiles for (A**)** CIP and (B) 4-NP. Pseudo-first-order photodegradation kinetics of (C) CIP and (D) 4-NP. (E) TOC removal efficiency and (F) scavenger tests using the AC and ACE25 samples.

Based on the previous results regarding stability, efficiency, and kinetics, the photodegradation of CIP and 4-NP was conducted using the AC and AC025 samples to analyze the enhancement caused by Eu^3+^ compared to the pure sample. The variations in the concentration of CIP and 4-NP (*C_n_*/*C*_0_) with respect to the exposure time to irradiation are shown in Figure 5A,B, respectively. Here, *C_n_* represents the absorbance after the irradiation time and *C*_0_ represents the initial absorbance of these organic molecules. The adsorption rates for ACE25 at CIP and 4-NP were 35, and 33%, respectively. These rates stabilize after 30 min of the adsorption/desorption process in the dark. For CIP, the AC sample achieved a degradation efficiency of 35%, whereas the ACE25 sample exhibited a significantly higher photodegradation efficiency, reaching approximately 80% of the initial CIP. With regard to the photodegradation of 4-NP, final efficiencies of 58 and 64% were observed for the AC and ACE25 samples, respectively. Although the final difference in efficiency between the samples is only 6%, excluding the adsorption process reveals that the photocatalytic efficiency of the ACE25 sample is 21% higher than that of the AC sample. Figure 5C,D (−ln(*C_n_*/*C*_0_) vs time) displays the rate constant values of the CIP and 4-NP photodegradation for AC and ACE25 samples. The reaction kinetics were faster for RhB, CIP, and 4-NP, with values of 0.0384 ± 0.00003, 0.0082 ± 0.00071, and 0.0024 ± 0.00015 min^–1^, respectively. Compared to the AC sample, there was an increase in the rate constants of 26, 256, and 118% for RhB, CIP, and 4-NP, respectively.

Since a significant improvement was noted for the ACE25 sample, its potential for the mineralization of organic residues was analyzed through total organic carbon (TOC) measurements. Figure 5E results show that even with an efficiency of degrading the initial CIP and 4-NP by 80 and 64%, respectively, the mineralization of these residues, i.e., their transformation into CO_2_ and H_2_O, occurred at a proportion of 27 and 17% for CIP and 4-NP, respectively. These results demonstrate that Eu^3+^ doping, even in small concentrations, can indeed enhance the photocatalytic behavior of inorganic semiconductors, reducing the concentration of organic pollutants in wastewater.

To gain qualitative insights into the ACE25 sample photodegradation mechanism, scavenger tests were conducted in the RhB photodegradation (Figure 5F), employing the use of AO to capture h^+^, BQ to capture ^•^O_2_H, and TBA to capture ^•^OH.^44,45^ It is observed that the photocatalytic efficiency was significantly inhibited by the presence of AO and BQ, indicating that h^+^ and ^•^O_2_H species are the main reactive species involved in the photodegradation mechanism. The ^•^OH radical also contributes, albeit in smaller quantities. This pathway is enhanced in the ACE25 sample compared with the undoped sample. Thus, the photogenerated charge pairs (e^–^–h^+^) from the semiconductor light absorption interact instantly with H_2_O and O_2_ in the environment. The e^–^ is excited to the CB of Ag_2_CrO_4_, allowing the reduction of O_2_ to superoxide radical (^•^O_2_^–^), while h^+^ from the VB oxidizes H_2_O, giving rise to an ^•^OH and a proton (H^+^). This H^+^ interacts directly with the previously generated ^•^O_2_^–^, resulting in ^•^O_2_H. This result is in line with previous studies involving Ag_2_CrO_4_ as a photocatalyst.^18,20^

To complement the experimental results, DFT calculations on three model systems are performed to simulate the Eu^3+^ doping process in Ag_2_CrO_4_. Since the incorporation of Eu^3+^ into the Ag_2_CrO_4_ network entails the substitution of Eu^3+^ for Ag^+^ and the creation of two V_Ag_. Therefore, to preserve charge neutrality, two V_Ag_ were introduced for each Eu^3+^ substitution (see details in Figure S7). Detailed information on how the creation of these V_Ag_ and substitutions on each model system is presented in the Supporting Information. The calculated values of relative energies, lattice parameters, and bond length distances can be found in Tables S4 and S5. In the calculated Eu^3+^-doped Ag_2_CrO_4_ systems, the substitution process is more favorable at [AgO_6_] than [AgO_4_] cluster, consistent with the fact that lanthanide elements show a preference for a high coordination environment.^46^ Specifically, the substitution at the [AgO_4_] cluster provokes a larger structural distortion with the formation of a new hepta-coordinated [EuO_7_] cluster, generating V_Ag_ in the adjacent 4c site. This modification is accompanied by the appearance of [AgO_5_] clusters in the neighbor 4c sites (Figure S8).

**Figure 6:**
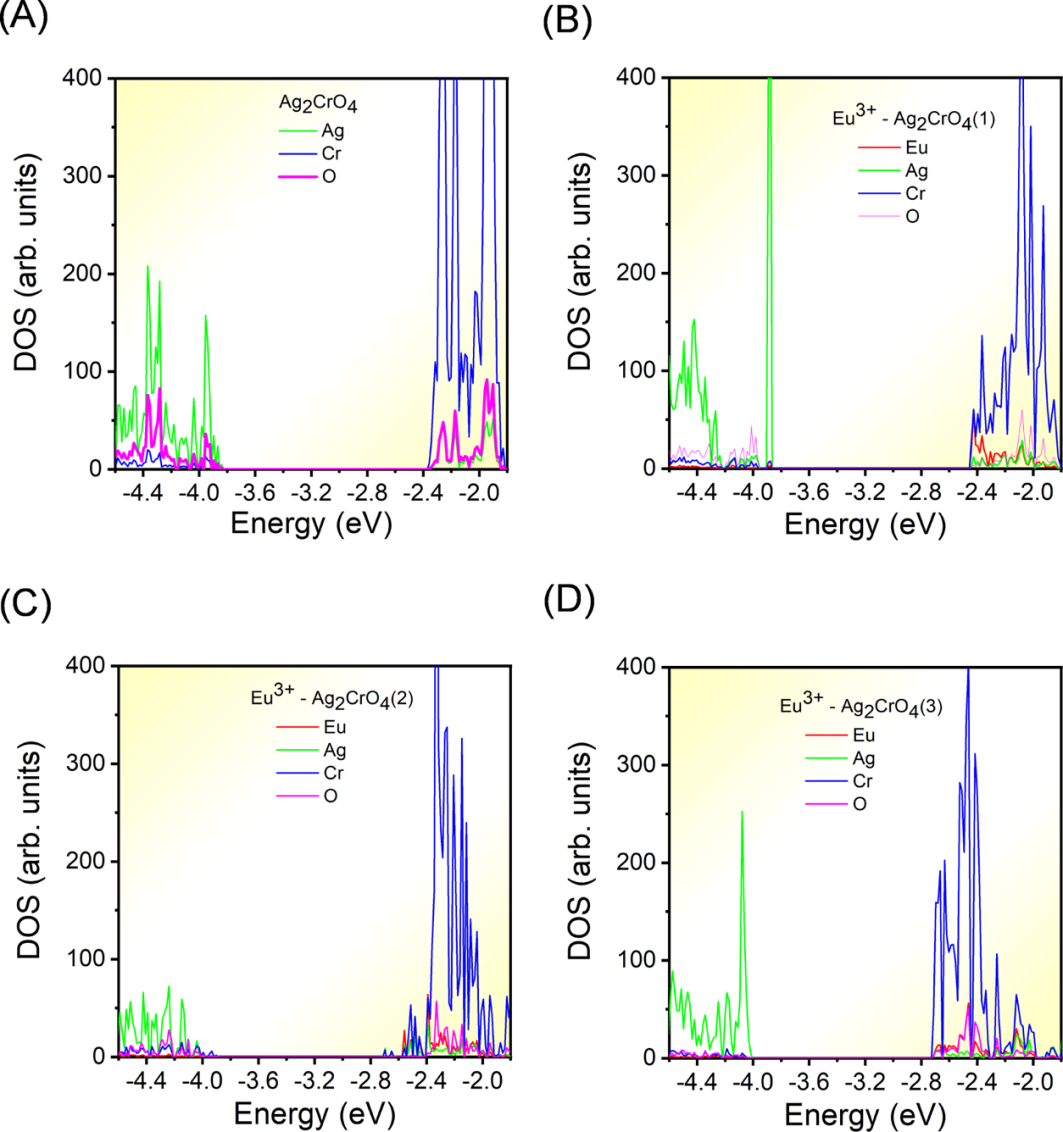
DOS projected on Ag, Cr, and O orbitals of (A) Ag_2_CrO_4_, (B) Eu^3+^-Ag_2_CrO_4_ substituted in [AgO_6_], (C) Eu^3+^-Ag_2_CrO_4_ substituted in [AgO_4_] and (D) Eu^3+^-Ag_2_CrO_4_ substituted in both [AgO_6_] and [AgO_4_].

Finally, we have performed an analysis of the density of states (DOS) for Ag_2_CrO_4_ and three modes systems. The top of the VB of Ag_2_CrO_4_ is mainly composed of Ag 4d and 2p orbitals, while the bottom of the CB is formed by the Cr 3d and 2p orbitals (Figure 6A). The calculated band gap is 1.46 eV lower than the experimental value, 1.71 eV. This discrepancy is due to the known underestimation of the band gap values calculated by the PBE functional. As shown in Figure 6B, Eu substitution at the [AgO_6_] cluster leads to a downward shift of the CB and VB, accompanied by a decrease in the band gap values from 1.46 to 1.41 eV. In the [AgO_4_] clusters, Eu^3+^ substitution results in the presence of new levels at the bottom of the CB due to the influence of Eu 4d states, causing a decrease in *E*_gap_ values from 1.46 to 1.30 eV, as depicted in Figure 6C. Finally, through the substitution of Ag^+^ with Eu^3+^ in both [AgO_6_] and [AgO_4_] clusters, the VB position is shifted by approximately −0.1 eV, and energy levels are introduced at the bottom of the CB, as illustrated in Figure 6D, with the value of *E*_gap_ being decreased from 1.46 to 1.18 eV. An analysis of Figure S9 shows a change in the nature from indirect to direct band gap in model (3).^47^ The analysis of the DOS reveals that Eu^3+^ doping in model (3) provokes a decrease of the *E*_gap_ value, as it has been previously observed in other ternary metal oxides during the Eu doping process.^48−50^ The most striking difference is that the Eu-4d states appear in the CB, while the defect state provoked by the V_Ag_ mainly contributes to the VB: in model (1), the contribution of tetrahedral Ag is notably increased compared to pure Ag_2_CrO_4_, while in model (2), there is a lower contribution of tetrahedral Ag by the formation of a [AgO_5_] coordination. We caution, however, that these comparisons of relative energies and DOS may not be entirely representative of the real conditions because of the higher theoretical values of % Eu^3+^ doping, 3.15 and 6.25%, with respect to experiments, 1%, so future computational studies on more realistic models are needed to study the complex behavior of Ag_2_CrO_4_-Eu^3+^-doped materials.

## 3 Conclusions

In the present study, the Eu^3+^ doping of the Ag_2_CrO_4_ lattice was implemented as an approach to address the environmental challenge posed by persistent pollutants. The main findings can be summarized as follows: (1) Eu^3+^ doping at low percentages was able to induce short-range symmetry breaking in the crystal lattice of Ag_2_CrO_4_, impacting its morphology; (2) the correlation between PL emissions and photocatalytic activity suggests a lower recombination rate resulting from the Eu^3+^ doping process; (3) the remarkable enhancement of stability and photocatalytic activity for the degradation of CIP and 4-NP was demonstrated by ACE25 sample; (4) scavenger experiments indicated ^•^O_2_^–^ and ^•^OH_2_ as the primary active species in the photodegradation mechanism. However, a secondary contribution of ^•^OH was found to be significant in enhancing the activity of the ACE25 sample; (5) DFT results reveal that the Eu^3+^ doping process induces Ag^+^ cation substitution in preferential octahedral sites of Ag_2_CrO_4_, leading to the formation of structural defects and new energy levels at the bottom of the CB. Furthermore, it affects band positions and reduces the *E*_gap_; (6) the modeling approach employed in this study may prove useful for future investigations targeting technological applications of Ag_2_CrO_4_ as a host material. In the context of the growing interest in developing visible-light-responsive photocatalysts, the innovative Eu-doped Ag_2_CrO_4_ systems introduced in this study contribute significantly to advancing sustainable wastewater treatment technologies. Additionally, understanding these systems lays the groundwork for further studies on the technological applications of other rare-earth-doped semiconductors.

## 4 Materials and Methods

### 4.1 Synthesis

For Ag_2_CrO_4_, 2 mmol of AgNO_3_ (99.8%, Cennabras) and 1 mmol of K_2_CrO_4_ (99.8%, J. T. Baker), solubilized separately in 0.05 L of deionized H_2_O, were heated to 60 °C until thermal stabilization. The resulting suspension was kept under heating and magnetic stirring for 10 min. The Eu^3+^ solution (1 mM) to obtain the doped samples was prepared by dissolving Eu_2_O_3_ in an acid solution, as previously described by our research group,^51^ maintaining the molar proportion and charge balance between Ag^+^ and Eu^3+^, corresponding to the ratio of 3Ag^+^/1Eu^3+^. Following this procedure, Ag_2_CrO_4_:*x*Eu, *x* = 0.0, 0.25, 0.5, and 1% in mol samples were prepared using the coprecipitation method. All samples were subsequently cooled to room temperature, and the precipitates were washed with deionized water and isopropyl alcohol, followed by drying at 50 °C for 12 h.

### 4.2 Photocatalysis

The photocatalytic performance of the samples was initially assessed through the degradation of 5 ppm of RhB solution (Aldrich, 95%) and 9 ppm of CIP (EMS, 99.8%) and 4-NP (Aldrich, ≥99%) solutions under visible light. In each experiment, the photocatalyst dosage was 1.0 mg/mL, and the resulting suspensions were submitted to an ultrasound bath and then transferred to an open reactor with a controlled temperature of 20 °C. In the absence of light, the suspension solution was homogenized for 30 min to reach the adsorption/desorption equilibrium. After, in the photocatalytic system, visible light was generated by six visible lamps (Philips TL-D, 15 W, 3.6 mW/cm^2^). During a specific interval, a 1 mL sample aliquot was collected, and subsequently, the catalyst particles were removed by centrifugation (1 × 10^4^ rpm for 5 min). The solutions were monitored by measuring their maximum absorption bands of these compounds (λ_RhB_ = 553 nm, λ_CIP_ = 276 nm, and λ_4-NP_ = 318 nm) using a V-660 UV-Vis absorption spectroscopy spectrophotometer (JASCO).

The stability of the photocatalytic activity is a crucial aspect of the photocatalyst quality. Therefore, recycling experiments were carried out on the degradation of RhB. Before each cycle, the samples were washed for dye removal, dried, and submitted to a new experiment. To the scavenger tests, equimolar amounts of ammonium oxalate monohydrate (AO) (Sigma-Aldrich, 99%), *tert*-butyl alcohol (TBA) (Sigma-Aldrich, 99.5%), and p-benzoquinone (HR) (Alfa-Aesar, 98%) were added to the RhB, CIP, and 4-NP solutions with the catalyst before irradiation as photogenerated holes (h^+^), hydroperoxyl radicals (^•^O_2_H), and hydroxyl radicals (^•^OH) scavengers, respectively. Total organic carbon concentration (TOC) was measured on a GE Sievers InnovOx analyzer by taking 30 mL of final and initial solutions. The TOC determination was carried out after mixing a diluted volume of the treated sample with H_3_PO_4_ (6 M) and Na_2_S_2_O_8_ (30% m/V) solutions for the determination of the inorganic and total carbon, respectively. The TOC content was analyzed by subtraction of the measured values of inorganic and total carbon, in terms of generated CO_2_.

### 4.3 Characterization Techniques

X-ray diffraction (XRD) analyses were performed using a diffractometer (Rigaku) with Cu Kα radiation (λ = 1.5406 Å). X-ray diffraction patterns were acquired with steps of 0.02° and a sweeping angular range between 10 and 110°. Raman spectra were collected using an iHR550 spectrometer (Horiba Jobin Yvon), coupled to an Ag ion laser (MellesGriot) operating at 633 nm with a maximum power of 200 mW and a fiber microscope in the range 100–1400 cm^–1^. UV–vis measurements were carried out on a Varian Cary 5G spectrometer in diffuse reflectance mode at room temperature in the 800–300 nm range. XPS analyses were performed on a Scientia Omicron ESCA spectrometer (Germany) using a monochromatic X-ray source of Al Kα (1486.7 eV). Peak deconvolution was performed using a 70:30% Gaussian–Lorentzian line shape and a Shirley nonlinear sigmoid-type baseline. The binding energies of all elements were calibrated with reference to the C 1s peak at 284.8 eV. The morphology of the samples was analyzed through images obtained using a scanning electron microscope Zeiss-Supra 35 with secondary electron detector (ETD, Everhart–Thornley detector). Particle size was measured using the linear method in ImageJ software from the obtained images. Photoluminescence spectra (PL) were measured using a laser (Cobolt/Zouk; λ = 355 nm) was used as the excitation source with 50 μW of incident potency and focused on the 20 μm position which at 298 K excited the samples. A 20 cm spectrometer (Andor Technologies) with the signal detected by a charge-coupled device detector was used to obtain the backscattered luminescence.

### 4.4 Computational Methods and Model Systems

First-principles calculations within the periodic DFT framework, employing the Perdew-Burke-Ernzerh (PBE) functional,^52^ were performed using the CRYSTAL17 program^53^ to characterize the Ag_2_CrO_4_ and Eu^3+^-doped Ag_2_CrO_4_ samples. All-electron basis sets were used to describe O,^54^ Ag,^55^ Cr,^56^ and atomic centers, while a 4f-in-core pseudopotential was used for the Eu atom.^57^ Regarding density matrix diagonalization, the reciprocal space net was described by a shrinking factor of 4, corresponding to 36 k-points generated according to the Monkhorst–Pack scheme. The accuracy of the evaluation of the Coulomb and exchange series was controlled by five thresholds: Coulomb overlap tolerance, Coulomb penetration tolerance, exchange overlap tolerance, exchange pseudooverlap in the direct space, and exchange pseudooverlap in the reciprocal space, whose adopted values were 10^–8^, 10^–8^, 10^–8^, 10^–8^, and 10^–16^, respectively. The equilibrium configuration was determined by minimizing the static lattice energy according to the Newton–Raphson method.^58,59^ At each iteration, the inverse matrix of the second derivatives of the energy was updated using the BFGS relation.^60^ The Raman vibrational modes and their corresponding frequencies were calculated from the undoped and Eu^3+^-doped Ag_2_CrO_4_ unit cell, using numerical second derivatives of total energies.

A supercell 2 × 2 × 1 composed of 112 atoms (comprising 32 Ag, 16 Cr, 64 O) was selected to simulate the Ag_2_CrO_4_ structure and the Eu^3+^ doping was performed at Ag^+^ cations positions. As octahedral and tetrahedral sites are available for Ag^+^ occupation in the Ag_2_CrO_4_, initially, a single Eu substitution was accomplished for each site corresponding to a doping percentage of 3.12%. In order to preserve charge neutrality, given the distinct charges between Ag^+^ and Eu^3+^ ions, two Ag^+^ vacancies (V_Ag_) were created for each Eu^3+^ substitution. When two Ag^+^ cations are substituted by two Eu^3+^ cations, a doping percentage of 6.25% was generated. Three model systems were selected to simulate the substitution processes: (1) two substitutions in [AgO_6_] clusters, (2) two substitutions in [AgO_4_] clusters, and (3) one substitution in the [AgO_6_] cluster and another at the [AgO_4_] cluster (only for 6.25%). In these models, two V_Ag_’s are generated in the adjacent [AgO_4_], [AgO_6_], [AgO_4_], and [AgO_6_] clusters, respectively (Figure S7).

The relative stability of Eu^3+^-doped Ag_2_CrO_4_, 6.25 and 3.15%, with respect the undoped Ag_2_CrO_4_ structure was calculated following eqs 1 and 2, respectively:
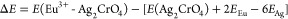
1
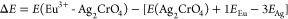
2where *E*(Eu^3+^-Ag_2_CrO_4_) and *E*(Ag_2_CrO_4_) are the total energies of the doped and undoped systems, respectively, and *E*_Eu_ and *E*_Ag_ are the total energies of Eu^3+^ and Ag^+^, respectively.

## Supporting Information Available

Comprehensive structural and electronic characterization data of the prepared samples, including Rietveld Refinement, PL spectra, and UV–vis spectroscopy; representations and data of the simulated systems; theoretical Raman spectra; and comparative analysis of XRD and FE-SEM characterizations of the samples before and after the fifth recycling run (PDF)
