# Supplementary material for: Boosted Photocatalytic Activities of Ag2CrO4 through Eu3+-Doping Process
Source: ACS Omega. 2024 Aug 7;9(33):35537–47. doi: 10.1021/acsomega.4c02683 (PMC11339825; doi:10.1021/acsomega.4c02683)
Supplement: Supplementary file 1 — ao4c02683_si_001.pdf [file ao4c02683_si_001.pdf]

Supporting Information:

Boosted photocatalytic activities of Ag_2CrO_4 through Eu^{3+}-doping process

Josiane C. Souza[1,2*], Samantha C. S. Lemos[2], Marcelo Assis[2], Carlos H. M. Fernandes[1], Lara K. Ribeiro[1,2], Yeison Núñez-de la Rosa[3,4], Márcio D. Teodoro[5], Lourdes Gracia[2,6], Juan Andrés[2*], Lucia H. Mascaro[1], Elson Longo[1*]

[1]CDMF, Federal University of São Carlos (UFSCar), São Carlos, 13565-905, Brazil.

[2]Department of Physical and Analytical Chemistry, University Jaume I (UJI), Castelló 12071, Spain.

[3]Department of Chemistry, Federal University of São Carlos (UFSCar), São Carlos, 13565-905, Brazil.

[4]Faculty of Engineering and Basic Sciences, Fundación Universitaria Los Libertadores, Bogotá, 111221, Colombia.

[5]Department of Physics, Federal University of São Carlos (UFSCar), São Carlos, 13565-905, Brazil.

[6]Department of Physical Chemistry, University of Valencia, Valencia, 46010, Spain.

*Corresponding author: josi3souza@gmail.com; elson.liec@gmail.com; andres@qfa.uji.es

Table S1. Cell parameters and quality indicatives obtained from the Rietveld method of the as-synthesized samples.

Sample	Lattice Parameters (Å)			V (Å³)	χ^2	R_p (%)	R_{wp} (%)
	a	b	c				
**AC**	10.0640	7.0220	5.5382	391.38	1.60	11.43	17.55
**ACE25**	10.0678	7.0280	5.5401	392.00	1.35	10.20	14.43
**ACE50**	9.9989	6.9830	5.5026	384.21	1.95	14.0	19.0
**ACE100**	10.0567	7.0268	5.5322	390.94	1.45	10.8	15.5

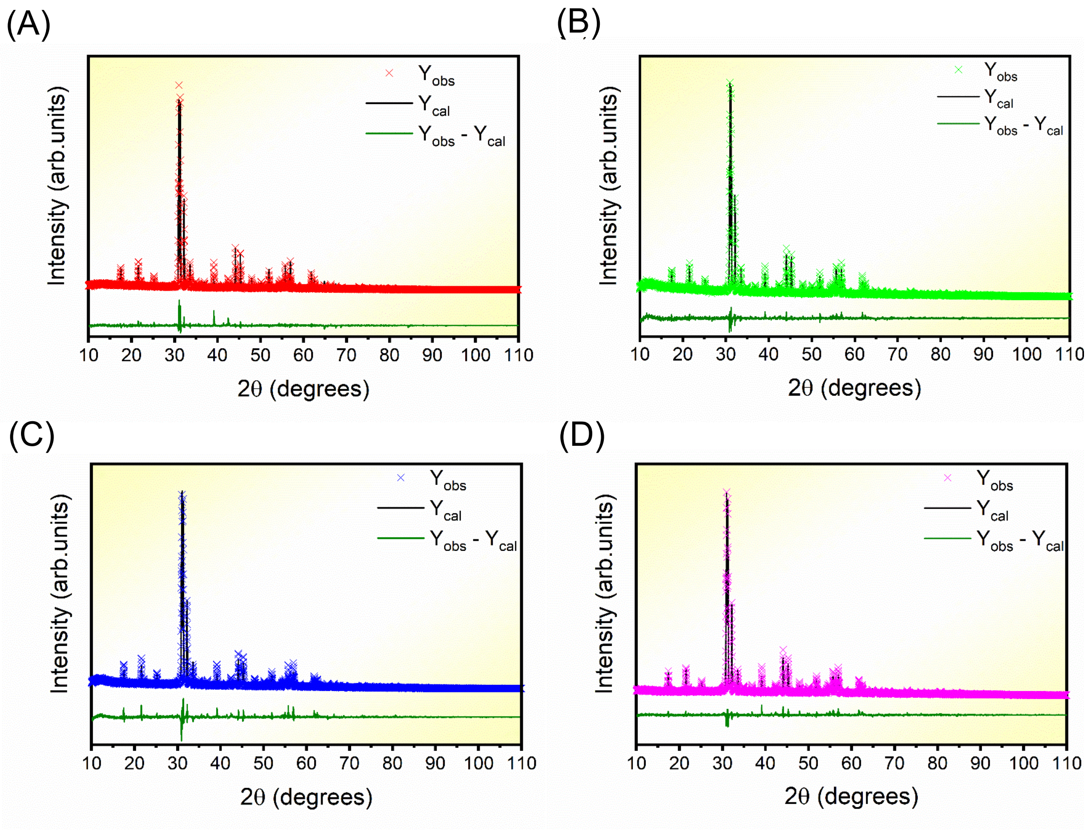

Figure S1. Rietveld refinement plot of the AC (A), ACE25 (B), ACE50 (C), ACE100 (D) samples.

Table S2. Reported values of the lattice parameters of Ag_2CrO_4 (Crystal Structure Database (ICSD).

Number ICSD	Lattice Parameters (Å)			V (Å³)	Structure	Spacial group
	a	b	c			
**252779** [1]	10.0667	7.0252	5.5405	391.83	orthorhombic	Pnma
**29580** [2]	10.0660	7.0200	5.5360	391.00	orthorhombic	Pnma
**16298** [3]	10.0630	7.0290	5.5400	391.86	orthorhombic	Pnma

We have calculated the Raman spectra of Ag_2CrO_4 in a single, in which the Eu^{3+} doping percentage is 12.5 %. The calculated Raman spectra of Ag_2CrO_4 unit cell are displayed in **Figure S2**. Two intense A_g modes, associated to the symmetrical stretching of Cr–O bonds of in [CrO_4] tetrahedra, at 834 and 858 cm^{-1} are sensed. The low-intensity bands in the region between 350 and 410 cm^{-1} are assigned to symmetrical and asymmetrical bending modes (A_g, B_{1g}, B_{2g}, B_{3g}) of O–Cr–O and Ag–O–Cr bond angles, and the weak B_{1g} mode observed at 223 cm^{-1} correspond to the stretching of Ag–O bonds in [AgO_4] tetrahedra. The analysis of **Figure S2** reveals that the Eu^{3+} doping in the Ag_2CrO_4 unit cell alters the Raman spectra features. The specific impact on the spectrum profile depends on the site of Eu^{3+} occupation. As result of the Eu^{3+} substitution, the most intense band (858 cm^{-1}) shifts to lower energy. In the spectra of the 4a site Eu-doped Ag_2CrO_4 two weak A_g modes are observed at 222 and 234 cm^{-1} corresponding to the stretching of Ag–O and Eu-O bonds (B_{1g} mode in pure Ag_2CrO_4). Additional contribution is observed regarding to Eu–O–Cr stretching and Cr–O–Ag bending at 263 cm^{-1}. Low-intensity bands, assigned to symmetrical and asymmetrical bending modes, Cr–O–Eu bending, in the region between 352 and 439 cm^{-1} are found. Finally, important contributions in the range of 845 to 895 cm^{-1} are observed attributed to the bending and stretching of Ag–O–Cr and of Cr–O bonds, being the more intense at 845, 861, 885, 903 and 995 cm^{-1} (all with A_g symmetry). For the Eu-doped Ag_2CrO_4 in the 4c site, several weak A_g modes from 202 to 281 cm^{-1} are observed corresponding to the stretching of Ag–O and Eu-O bonds. Additional contribution is observed regarding to Eu–O–Cr stretching and Cr–O–Ag bending at 328 cm^{-1} and low-intensity bands, associated to symmetrical and asymmetrical bending modes of Cr–O–Eu, are found in the region between 353 and

456 cm^{-1}. Finally, intense bands attributed to Cr–O stretching bond are obtained at 829, 911 and 982 cm^{-1}. At this point, a note of caution is mandatory, the theoretical Raman spectra has calculated for a 12.5% of Eu^{3+} doping, which is much higher than the experimental value of 1%.

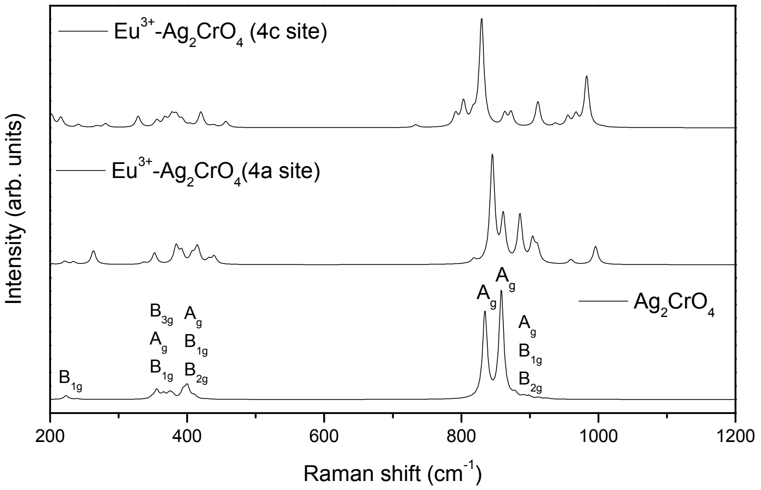

Figure S2. Theoretical Raman spectra of Ag_2CrO_4 and Eu^{3+}-doped Ag_2CrO_4 (resulting in a Eu^{3+} doping percentage of 12.5%).

Table S3. FWHM values obtained from the Raman band located at 807 cm^{-1} for the as-synthesized samples.

Samples	FWHM
**AC**	8.7
**ACE25**	18.5
**ACE50**	16.8
**ACE100**	16.0

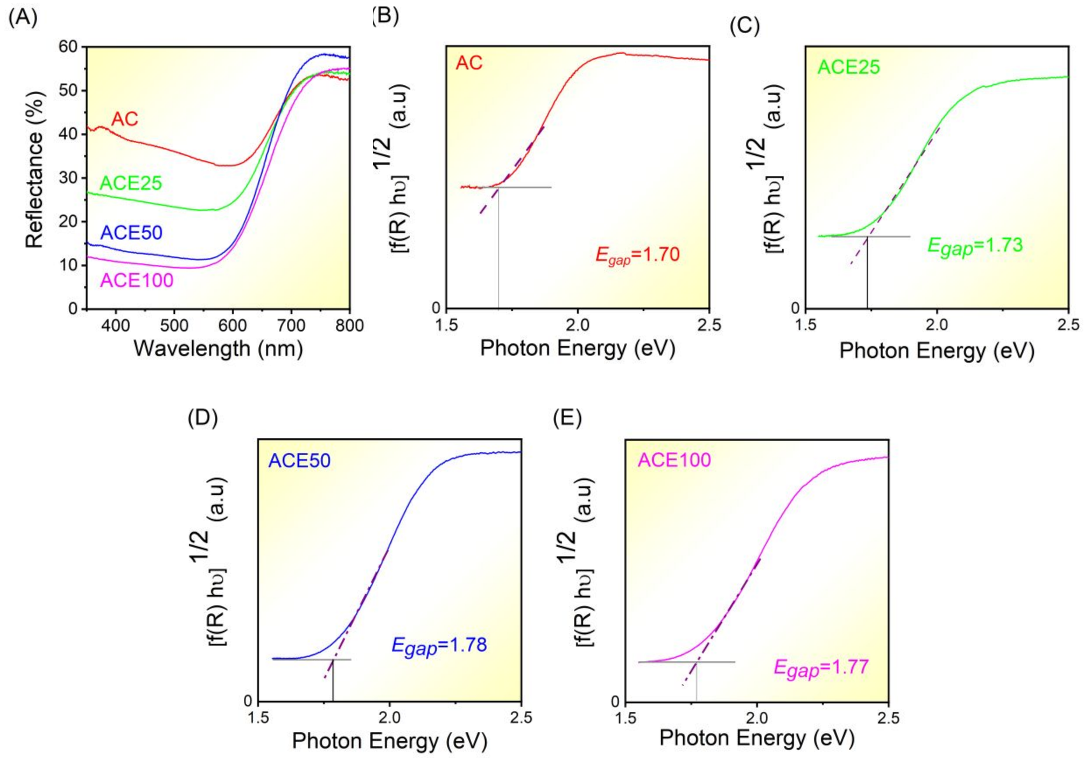

Figure S3. (A) UV-vis DRS of the samples, (B-E) Tauc plots $[F(R\infty)\cdot h\nu]^{1/2}$ vs hν (proton energy. The purple dashed lines correspond to linear extrapolations to determine indirect Egap values. The thin dark-gray lines correspond to sloped baselines extrapolated from the hν < 1.7 eV range.

In the present work, the prepared Ag_2CrO_4 samples show contributions to its baseline from the apparent absorption tail (see **Figures S3B-S3E**). To determine the band gap, a tangent (thin dark-gray) was first drawn to the baseline at low energies, hν < 1.7 eV (and a second line tangent (purple dashed) to the slope in the linear region of the absorption onset was drawn. The intersection of the two lines corresponds to the energy of the band gap. (see references: Chen Z., et al [4] and Makuta P., et al. [5]).

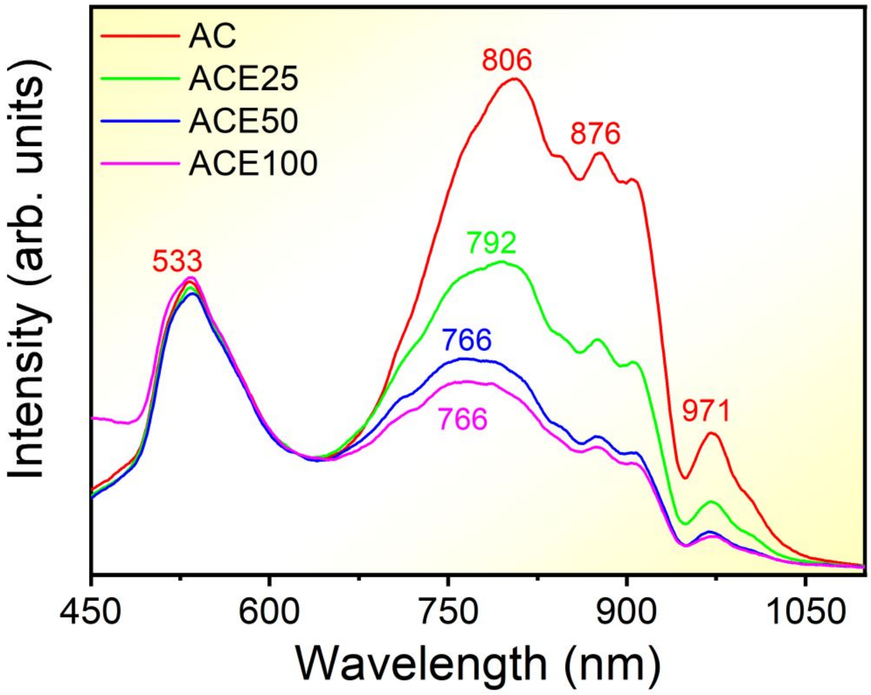

Figure S4. PL emission spectra of the AC, ACE25, ACE50, ACE100 samples.

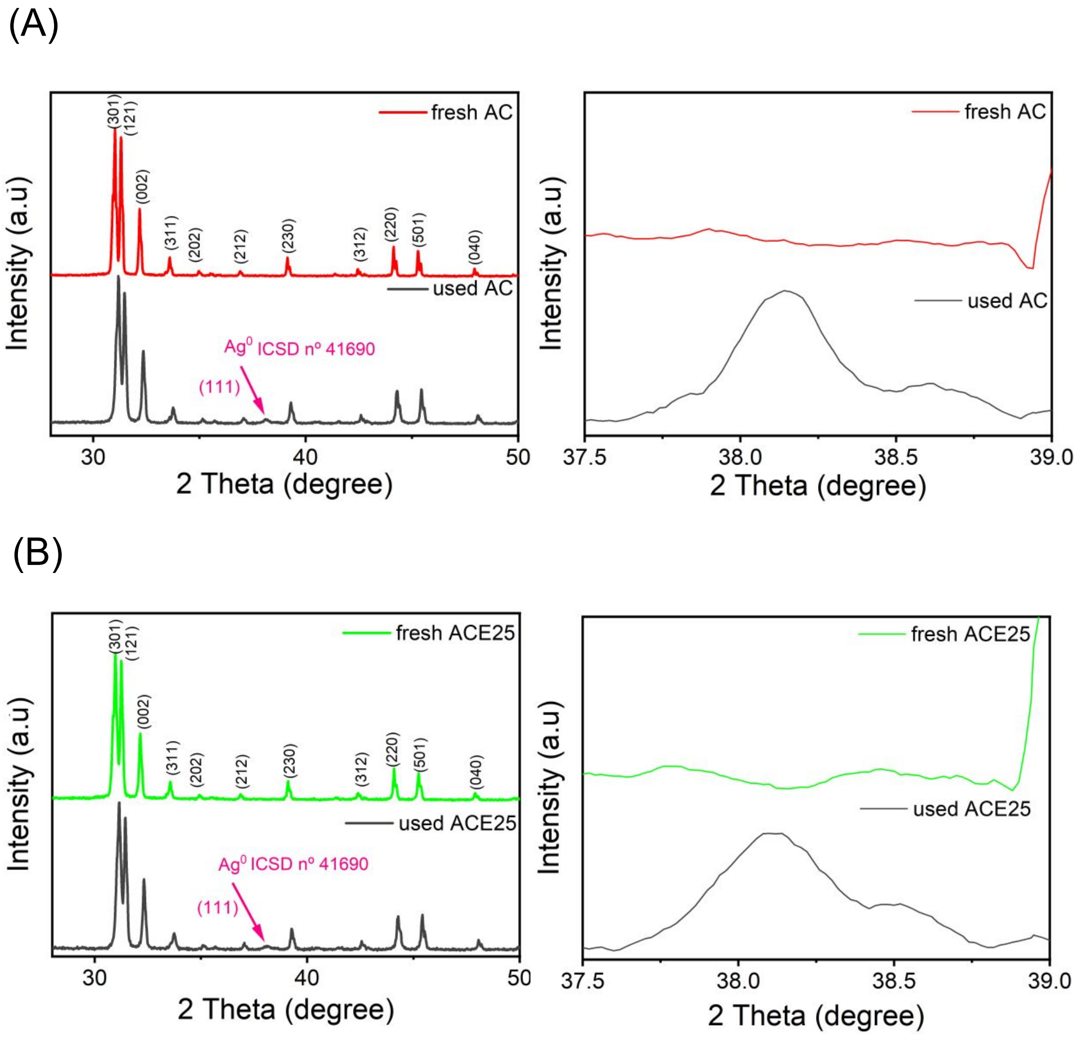

Figure S5. XRD patterns before and after fifth recycling run. (A) AC sample and (B) ACE25 sample (in the left). Zoom at XRD patterns within the 2θ range of 37.5º-39º for AC (A) and ACE25 (B) (in the right).

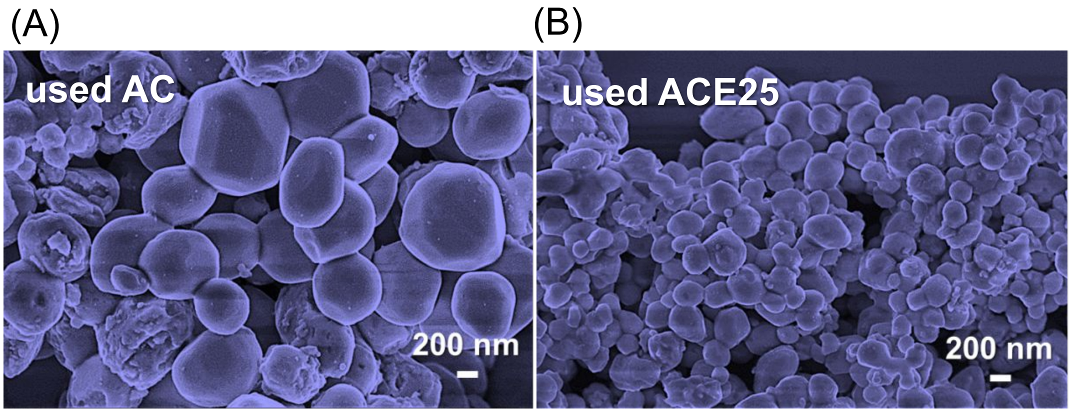

Figure S6. FE-SEM images after fifth recycling run. (A) AC sample and (B) ACE25 sample.

Table S4. Relative values, ΔE, for 3.12% and 6.25% Eu^{3+}-doped Ag_2CrO_4, considering as reference the model (1) as the most stable. Lattice parameters and volume of the cell is included for the three models.

**Theoretical systems**		**ΔE (eV)**	**Lattice parameters**			
			a (Å)	b (Å)	c (Å)	V (Å^3)
**3.12%**	Eu^{3+}-Ag_2CrO_4(1)	0.00	10.2028	7.1400	5.0985	371.4154
	Eu^{3+}-Ag_2CrO_4(2)	0.84	10.1891	7.1570	5.1491	375.4853
**6.25%**	Eu^{3+}-Ag_2CrO_4(1)	0.00	10.2047	7.1176	5.1173	371.6847
	Eu^{3+}-Ag_2CrO_4(2)	1.89	10.3673	7.2213	5.1419	384.9503
	Eu^{3+}-Ag_2CrO_4(3)	1.07	10.2971	7.1828	5.1250	379.0553

Table S5. Wyckoff position of Ag and Eu and Ag-O and Eu-O bond length for Ag_2CrO_4 and 6.25% Eu^{3+}-doped Ag_2CrO_4 systems. (1) Two Ag^+ atoms substituted by two Eu^{3+} atoms in 4a positions, (2) in 4c positions and (3) in 4a an 4c positions.

	**Ag_2CrO_4**		**Eu^{3+} doped Ag_2CrO_4**									
			(1)			(2)			(3)			
**Wyckoff**	Ag(4a)	Ag (4c)	Eu (4a)	Ag (4a)	Ag (4c)	Eu (4c)	Ag (4a)	Ag (4c)	Eu (4a)	Eu (4c)	Ag (4a)	Ag (4c)
**Ag-O/ Eu-O (Å)**	2.347	2.326	2.309	2.315	2.335	2.311	2.322	2.334	2.290	2.280	2.338	2.318
	2.347	2.326	2.309	2.333	2.365	2.322	2.349	2.357	2.317	2.302	2.377	2.332
	2.371	2.437	2.347	2.373	2.446	2.347	2.389	2.434	2.390	2.378	2.397	2.419
	2.371	2.697	2.347	2.393	2.656	2.475	2.433	2.570	2.397	2.445	2.418	2.569
	2.551		2.365	2.549		2.486	2.504	2.674	2.399	2.492	2.540	2.694
	2.551		2.365	2.559		2.488	2.629		2.403	2.532	2.634	
						2.582				2.596		

Based on the three models showed in **Figure S7,** the values of ΔE were calculated and compared. An analysis of the ΔE values render that that the substitution in the octahedral coordination site is energetically favored over the tetrahedral one at 3.12% and at 6.25% (by 0.84 eV and 1.89 eV, respectively), being the stability order (1)>(3)>(2) , as can be seen in **Table S4**.

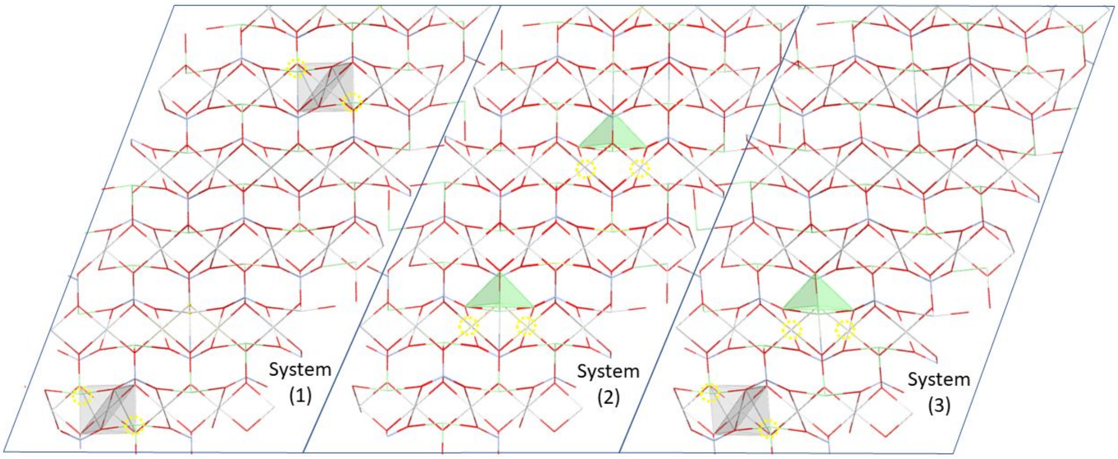

Figure S7. Representation of the three Eu^{3+} doping Ag_2CrO_4 systems simulated in this study. The gray polyhedra indicate the $[AgO_6]$ substitution site, the green ones the $[AgO_4]$ substitution site and the dotted yellow circles indicated the position of V_{Ag} formation.

An analysis of the results show that the extent of structural distortion depends on the substitution site. In model system (1), the substitution in 4c sites leads to a decrease of Eu-O bond lengths with respect Ag-O bonds, as a result, to a smaller lattice parameters and unit cell volume. Conversely, in the case of model system (2) where substitution occurs in the 4a site a slight increase of the Eu-O bond distances, and the lattice parameters and volume are verified. In case (3) the Eu-O bond lengths suffer both a small increase or decrease.

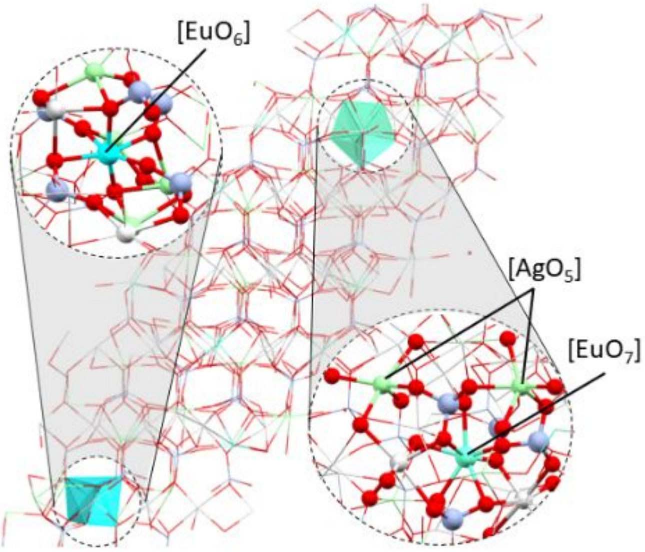

Figure S8. Illustration of the local coordination of the Eu^{3+} doped site in the Ag_2CrO_4 model system (3): [EuO_6] formation for substitution in 4a site and the [EuO_7] and [AgO_5] for substitution in 4c site.

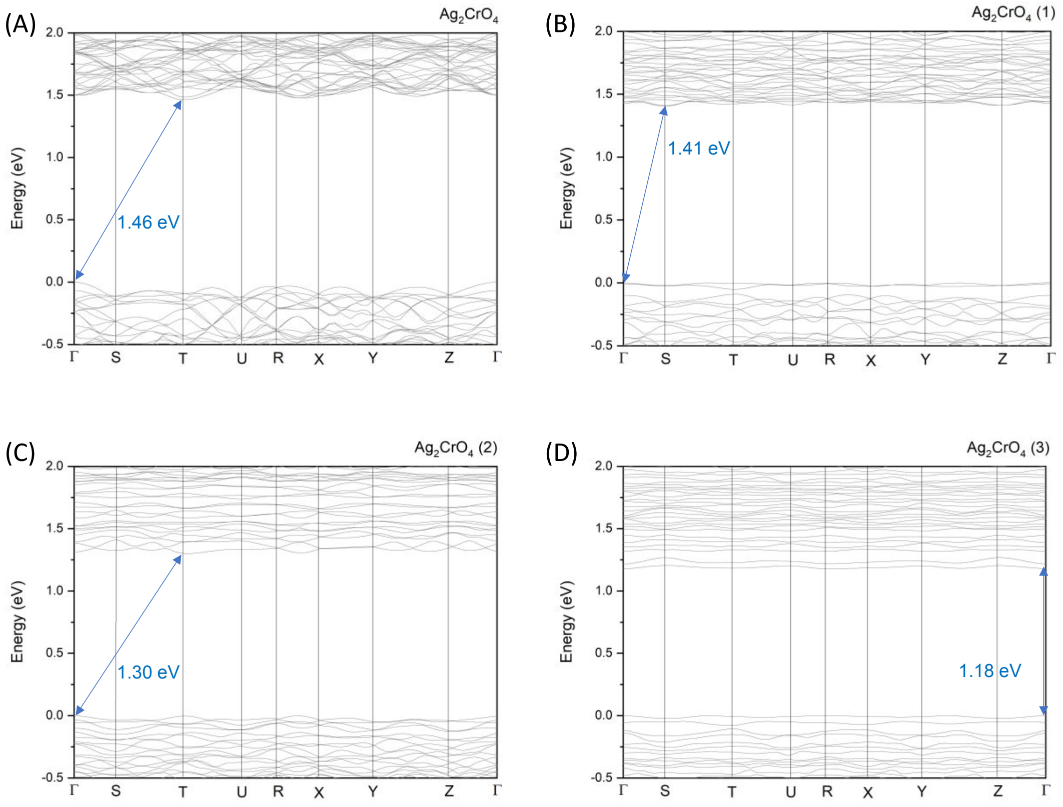

Figure S9. Band structures of (A) Ag_2CrO_4, (B) Eu^{3+}-Ag_2CrO_4 substituted in [AgO_6], (C) Eu^{3+}-Ag_2CrO_4 substituted in [AgO_4] and (D) Eu^{3+}-Ag_2CrO_4 substituted in both [AgO_6] and [AgO_4].

REFERENCES

(1) Fabbro, M. T.; Gracia, L.; Silva, G. S.; Santos, L. P. S.; Andrés, J.; Cordoncillo, E.; Longo, E. Understanding the Formation and Growth of Ag Nanoparticles on Silver Chromate Induced by Electron Irradiation in Electron Microscope: A Combined Experimental and Theoretical Study. *J. Solid State Chem.* **2016**, *239*, 220–227.

(2) Pinatti, I. M.; Tello, A. C. M.; Trench, A. B.; de Foggi, C. C.; Pereira, P. F. S.; Teixeira, M. M.; Jacomaci, N.; Andrés, J.; Longo, E. Zinc-Substituted Ag_2CrO_4: A Material with Enhanced Photocatalytic and Biological Activity. *J. Alloys Compd.* **2020**, *835*, 155315.

(3) Hackert, M. L.; Jacobson, R. A. The Crystal Structure of Silver Chromate. *J. Solid State Chem.* **1971**, *3*, 364–368.

(4) Chen, Z.; Miller, E.; Dinh, H. N. *Photoelectrochemical Water Splitting Standards, Experimental Methods, and Protocols*; Springer: New York, NY, 2013, 57.

(5) Makuła, P.; Pacia, M.; Macyk, W. How To Correctly Determine the Band Gap Energy of Modified Semiconductor Photocatalysts Based on UV-Vis Spectra. *J. Phys. Chem. Lett.* **2018**, *9*, 6814–6817.
